# Prediction of specificity-determining residues for small-molecule kinase inhibitors

**DOI:** 10.1186/1471-2105-9-491

**Published:** 2008-11-25

**Authors:** Daniel R Caffrey, Elizabeth A Lunney, Deborah J Moshinsky

**Affiliations:** 1 Pfizer Research Technology Center, 620 Memorial Drive, Cambridge, MA 02139, USA; 2 Pfizer Global Research and Development, 10777 Science Center Drive, San Diego, CA 92121, USA

## Abstract

**Background:**

Designing small-molecule kinase inhibitors with desirable selectivity profiles is a major challenge in drug discovery. A high-throughput screen for inhibitors of a given kinase will typically yield many compounds that inhibit more than one kinase. A series of chemical modifications are usually required before a compound exhibits an acceptable selectivity profile. Rationalizing the selectivity profile for a small-molecule inhibitor in terms of the specificity-determining kinase residues for that molecule can be an important step toward the goal of developing selective kinase inhibitors.

**Results:**

Here we describe S-Filter, a method that combines sequence and structural information to predict specificity-determining residues for a small molecule and its kinase selectivity profile. Analysis was performed on seven selective kinase inhibitors where a structural basis for selectivity is known. S-Filter correctly predicts specificity determinants that were described by independent groups. S-Filter also predicts a number of novel specificity determinants that can often be justified by further structural comparison.

**Conclusion:**

S-Filter is a valuable tool for analyzing kinase selectivity profiles. The method identifies potential specificity determinants that are not readily apparent, and provokes further investigation at the structural level.

## Background

The human genome contains approximately 500 protein kinases that regulate numerous cellular processes via protein phosphorylation [1]. Protein kinases mediate cell signaling pathways that are important for metabolism, development, apoptosis, immune responses, cell proliferation, and differentiation. Several of these pathways have been implicated in cancer, inflammation, and metabolic diseases. Thus, a number of protein kinases have been proposed as drug targets for these diseases [2]. Designing selective kinase inhibitors is a major challenge in drug discovery and development. The gene family is large and most kinases domains are similar in sequence and structure. The selectivity issues associated with small molecules that bind to the ATP catalytic binding site are particularly challenging as most kinases have the same active-site chemistry.

Understanding the basis of kinase inhibitor selectivity is crucial to the design of safe and efficacious drugs. Ideally, a drug will inhibit a small set of kinases that are relevant to the disease while avoiding the inhibition of kinases that may lead to toxic side effects. For example, imatinib inhibits a number of kinases that are believed to be important for certain cancer types [3]. However, most kinase-targeted drugs exhibit one or more toxic side effects that may include skin rash, gastrointestinal perforation, diarrhea, vomiting, cardiotoxicity, and bleeding [4,5]. To avoid potential toxic side effects, most kinase drug discovery projects assess the selectivity of their small molecules against a panel of kinases. Typically, drug discovery teams follow up on lead compounds that inhibit a small number of kinases with the goal of further optimizing selectivity along with other pharmacokinetic properties.

There are at least two major challenges associated with selectivity optimization: 1) understanding the basis for the measured selectivity profile and how it might be improved, and 2) extrapolating from the measured profile to the rest of the kinome as complete selectivity data are rarely available. Although this work is primarily concerned with first task, the two problems are not always considered separately. Indeed, several studies have focused on variations of these two problems by exploring relationships between sequence, structure and small molecule selectivity [6-10]. Vulpetti *et al *identified the most variable residues in the ATP binding site as good interaction sites for specific inhibitors. It was shown that kinases with less than 60% sequence identity are poorly correlated with SAR similarity [6,7]. In contrast, kinases with greater than 60% identity have a good chance of being inhibited by the same set of compounds.

Unfortunately, these observations do not translate into accurate prediction of kinase off-targets, i.e., those kinases other than the intended kinase that are inhibited. Sheinerman *et al *[8] also evaluated sequence identity as a predictor of kinase off-targets. For example, when the kinase off-targets for a given inhibitor are predicted to be those for which fewer than seven binding site residues are non-identical, only half of genuine off-targets were predicted correctly [8]. The sensitivity (i.e. the number of correctly predicted off-targets divided by the total number of known off-targets) of this prediction was improved to 0.69 by setting the threshold to eleven non-identical binding site residues. However, this was detrimental to the specificity of the prediction, as less than half of non-targets were correctly predicted as non-targets. By restricting analyses to energetically important binding site residues, Sheinerman *et al *were able to improve the sensitivity and specificity of off-target predictions.

Following these studies, we hypothesized that metrics such as sequence identity may be too general to explain selectivity data. For example, p38α, p38β, p38γ, and p38δ all belong to the same subfamily and their binding sites are very similar in sequence. However, a single residue difference (Met to Thr) appears to be sufficient for some compounds to distinguish p38α and p38β from p38γ and p38δ [11]. Furthermore, more distantly related kinases (e.g. NLK and GAK) that have the same specificity determining threonine are also inhibited by the same compound [12]. This suggests that single-residue differences need to be considered independently when attempting to explain kinase selectivity.

It is also clear that kinase selectivity needs to be rationalized in the context of three-dimensional co-crystal structures. Although structures are not available for every kinase, it is common to dock a compound from a solved X-ray structure to other superposed kinase structures. The docking procedure can be performed manually or with the aid of standard docking and energy minimizing programs [13]. Rationalizing selectivity across multiple docked kinase structures requires careful assessment of the docked poses. Proposed steric clashes may point to a *bona fide *selectivity determining residue or an erroneous kinase – small molecule conformation.

Recognizing the limitations of sequence identity as a predictor of kinase off-targets and the need to consider structural information, we have developed a complementary approach known as S-Filter. S-Filter relies on structural and sequence information to predict specificity determinants for a particular kinase inhibition profile. It is intended as a hypothesis generation tool that will prompt further investigation at the structural level. Here, we describe S-Filter and its application to kinase selectivity data.

## Results

A novel method called S-Filter was developed to predict specificity-determining residues for kinase inhibitors. The method is based on the assumption that a compound's affinity for one set of kinases over another set of kinases is due to the presence of residues that either permit or prevent binding, respectively. Intuitively, we expect a specificity determinant to be one or more residues that are primarily found in the set of inhibited kinases. It also seems reasonable to assume that the set of inhibited kinases will have identical or very similar residues at the site of specificity. That is, the specificity determinants will be conserved in sequence across the inhibited kinases. Furthermore, specific compounds often fill a small cavity that is unique to the set of inhibited kinases. Kinases that have a bulkier residue at the corresponding position will not have a cavity that can be occupied by the compound. Instead, the compound will clash with the bulkier residue, thus preventing potent inhibition. This suggests that specificity determinants will often be relatively small residues. Of course, the presence of a bulkier residue does always lead to an unfavorable steric clash. A large residue can often adopt a different conformation that accommodates the compound. However, a large residue is less likely to accommodate a compound when it is buried deep in the confines of the active site pocket where there is little room to maneuver. This suggests that specificity determining-residues will often have low solvent accessibility.

We therefore consider four key questions when predicting specificity-determining residues: 1) How specific is the binding site residue to the set of inhibited kinases? 2) Is the residue deep in the pocket where steric clashes are less likely to be avoided? 3) Is the residue small and likely to create a sub-pocket that is unique to the set of inhibited kinases? 4) Is the residue conserved in sequence among the set of inhibited kinases? These four parameters are quantified by the N-score, SA-score, MW-score, and C-score respectively. Each of these terms contributes to a Filter-score and are described in more detail below.

### Residue Filters

Before we explain the finer details of S-Filter, it is necessary to describe how a residue filter behaves in PFAAT [14]. A reside filter allows one to only view sequences that have a particular residue at an alignment column. For example, one can imagine applying an alanine filter to column 10 of a kinase multiple sequence alignment. As a result, all kinase sequences that lacked an alanine at column 10 would not be displayed in the alignment; the visible kinase sequences would all have an alanine at column 10. If the set of visible kinases are potently inhibited by a small molecule, and the set of hidden kinases are not inhibited by the same small molecule, we have also applied a potential selectivity filter at column 10. In other words, a selectivity filter is a residue filter that is applied to a specificity determining residue.

### S-Filter

Given a set of alignment columns that correspond to a small-molecule binding site, S-Filter attempts to identify the set of alignment columns that best explain an inhibition profile. To do this, S-Filter identifies a subset of residues that can act as a potential filter for the low-affinity kinases. S-Filter applies residue filters to one or more alignment columns until the set of uninhibited kinases are hidden from the display and only potently inhibited kinases remain visible. S-Filter typically applies filters to alignment columns that have residue(s) which distinguish the high-affinity kinases from the low-affinity kinases.

For example, the high affinity kinases may have a glycine at an alignment position where the low-affinity kinases have other residue types. By applying a filter for glycine, the set of uninhibited kinases would be filtered out. Thus, the set of applied filters are predicted as specificity-determining residues, as their combination will only be found in the kinases that bind the compound with high affinity.

The order in which residue filters are applied to alignment columns is determined by a Filter-score that is described below. To ensure that all the inhibited kinases remain visible, a residue filter must specify those residues that are present in the set of inhibited kinases. Obviously, a residue filter will have maximum effect when none of these residues are present in the set of uninhibited kinases.

S-Filter is implemented in PFAAT [14], a Java application which provides an interface to analyze and annotate multiple sequence alignments. S-Filter requires a selectivity profile for the compound of interest, a solved three-dimensional structure of the compound in complex with a kinase, and a multiple sequence alignment of the kinases in the selectivity profile. The selectivity data described below was loaded into PFAAT as sequence annotations. A threshold of 75% was set to distinguish the set of inhibited kinases from the set of uninhibited kinases. An exception was made for PHA-00781089, which inhibits MK2 at 70%.

### Filter-score

S-Filter computes a Filter-score for each binding site column in the multiple sequence alignment, and applies the residue filter to the highest scoring column. A high scoring Filter-score implies the column has one or all of the following: 1) A set of residues that are unique to the set of inhibited kinases as specified by the N-score, 2) A residue that is buried deep in the reference protein structure as specified by the SA-score. 3) Small residues within the set of inhibited kinases as specified by the MW-score. 4) A set of residues which are either identical or very similar among the set of inhibited kinases as specified by the C-score.

The filtering process is iterated until all uninhibited kinases are filtered out, or all possible columns have had a filter applied. The Filter-score for a given column is the product of the following terms:

Filter-score = N-score × SA-score × MW-score × C-score

The Filter-score is a simple heuristic that attempts to incorporate some of our basic assumptions and understanding of small molecule specificity. It does not attempt to incorporate other structural features that cannot be computed for every kinase in the selectivity panel.

### N-score

Residues that are unique to the set of inhibited residues are potential selectivity determinants. The objective of the N-score is to identify residues that are unique to the set of inhibited kinases. Therefore, S-Filter counts the number of kinases (N-score) below the inhibition threshold that do not have any of the residues that exist in the set of inhibited kinases. It is designed to place greater weight on alignment columns where the set of inhibited kinases have residues that are absent or rarely possessed by the set of uninhibited kinases. For example, if a compound inhibits two of the twenty seven kinases in our selectivity panel, a maximum N-score of twenty five would indicate that none of the uninhibited kinases possess the same residues that belong to the two inhibited kinases. Invariant binding site columns will be assigned a minimum N-score of zero as the set of uninhibited kinases will be identical to the set of inhibited kinases. For example, the glutamate in α helix C is identical across our panel of kinases and it is unlikely that it is directly involved in determining specificity for a small molecule.

### SA-score

Residues with low solvent accessibility are likely to be deep in the pocket and will often make significant interactions with the small molecule. Importantly, residues with low solvent accessibility are less likely to avoid steric clashes. Such residues will have difficulty maneuvering within the confines of the bind site and they are expected to have difficulty adopting different conformations. S-Filter computes a SA-score (100 minus relative solvent accessibility) that is designed to place greater weight on residues with low solvent accessibility. The relative solvent accessibility is computed from a crystal structure of a kinase belonging to the set of inhibited kinases. This allows S-Filter to incorporate information derived from a three dimensional structure without requiring a crystal structure to be solved for all kinases in the selectivity panel. An alignment column will have a high SA-score when its residue belonging to the reference structure has a low solvent accessibility value.

### MW-score

Small residues are likely to create a sub-pocket that is not found in kinases with a bulkier residue at the corresponding position. A bulkier residue is more likely to cause a steric clash with a nearby substituent. S-Filter computes a MW-score (the molecular weight of Trp, the largest residue minus the molecular weight of the largest residue in the set of inhibited kinases). The score is designed such that small residues will have higher scores. An alignment column will have a high MW-score when the set of inhibited kinases have small residues and the set of uninhibited kinases have larger residues at the corresponding position.

### C-score

To ensure that all the inhibited kinases remain visible, a residue filter must specify all residues that are present in the set of inhibited kinases. This set of residues could potentially include several different types of residue. In contrast, specificity determining residues are generally expected be identical or similar in physical chemical properties. The Von Neumann entropy conservation score (C-score) is used to up-weight conserved residues [14]. An alignment column will have a high C-score when the set of inhibited kinases have identical or similar residues at the corresponding position.

### Binding sites and residue accessibilities

The tree dimensional structures were used to define the binding sites and to compute relative residue solvent accessibilities in PFAAT [15]. Residue accessibilities were determined for each protein chain in the absence of its small molecule. The residue accessibilities were used to compute the SA-score above.

All residues that undergo a relative solvent accessibility change of 1% or more when bound to the small molecule of interest are defined as binding site residues. S-Filter analysis was applied to the alignment columns that correspond to binding site residues for the appropriate small molecule.

### Compound selection

**Figure 1:**
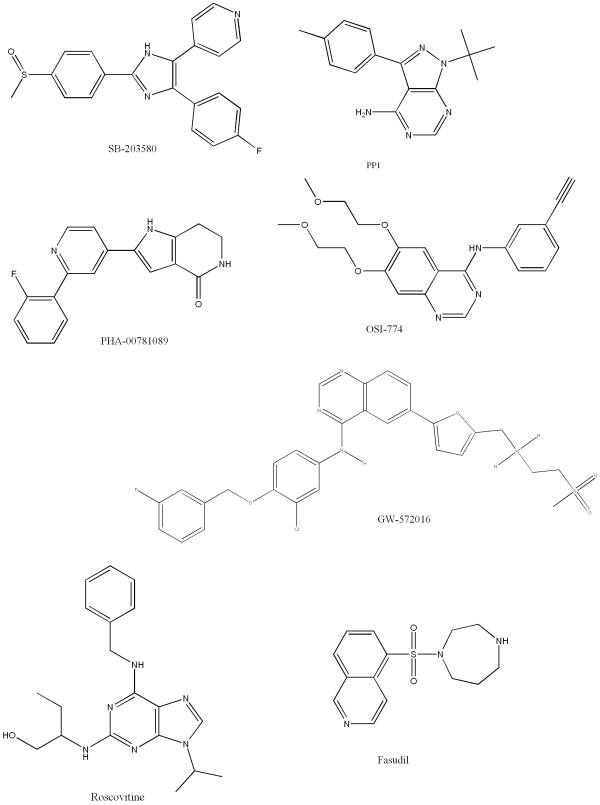
Small molecule kinase inhibitors analyzed in this work.

**Figure 2:**
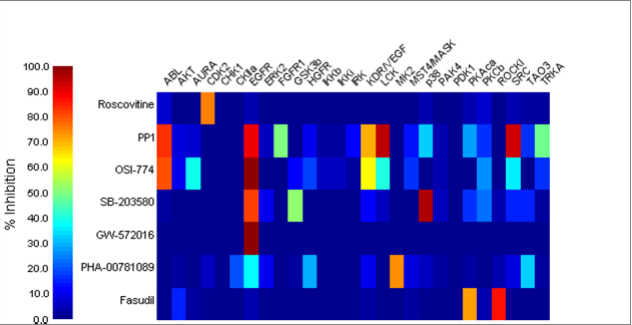
**Heatmap for the kinase selectivity profile**. Mean percent inhibition data for all compounds (1 μM) run in duplicate, are displayed as false colors according to the legend.

Seven kinase inhibitors (Figure 1) were tested in our kinase panel (Figure 2). The raw data for the kinase assays are provided in Additional file 1 and are also available at . The seven compounds were chosen because they have been determined to be selective in other kinase panels [12,16-18] and their three-dimensional structures have been solved in complex with a relevant kinase [11,19-24]. To ensure each compound belongs to a distinct chemical series, we require all compounds to have pair-wise Daylight^® ^fingerprint [25] Tanimoto scores less than 0.5. For the purpose of validating the method, it is desirable to select compounds where the selectivity determinants are described by independent research groups. Selectivity determinants were previously described for four of the seven compounds [11,20,22,24,26]. The PDB codes are listed in Table 1.

**Table 1:** S-Filter predictions

**Compound**	**Primary target**	**PDB**	**Predictions**	**FN**	**FP**	**TN**	**TP**	**Total**
SB-203580	p38	1A9U	*Leu 104 *Thr 106	0	0	14	2	16
PHA-00781089	MK2	2P3G	Cys 140 Gly 143	0	1	18	1	20
Roscovitine	CDK2	2A4L	Val 64 Leu 83 *Ala 144*	0	2	20	1	23
PP1	SRC/HCK	1QCF	Thr 338 Gly 344	0	1	18	1	20
OSI-774	EGFR	1M17	*Thr 766 *Cys 773	0	1	19	1	21
GW-572016	EGFR	1XKK	*Cys 775*	2	0	23	1	26
Fasudil	ROCK1	2ESM	Val 137 Met 153 Ala 215	1	2	14	1	17

Predicted specificity determinants are shown for each compound and are either designated as false positives (FP) or true positives (TP). Five of the predicted residues are in italics as they are self-designated true positives after validating them through structural superposition studies. Residues that were not predicted as specificity determinants are not shown but are either designated as false negatives or true negatives. The total number of residues in the binding site are shown in the final column.

### Assessing the predictions

S-Filter predictions were made for all seven compounds and are summarized in Table 1. To evaluate prediction accuracy, we compiled a list of residues that were proposed by independent groups to be specificity determinants. After careful examination of three-dimensional structures, we also concluded that four additional predictions should be treated as true positives. These self-designated true positive predictions are italicized in table 1 and are rationalized below. In the absence of supporting data, the remaining predictions were designated as false positives. In general, S-Filter does a reasonably good job of predicting specificity determinants (Table 1). The predictions are evaluated in more detail below.

### Kinase selectivity profile

All protein kinase assays (Table 2) were run in a 384-well format, using either a Caliper protocol [27,28] or a radioactive protocol [29,30]. Each compound was tested in duplicate at 1 μM to determine a percent inhibition value. The enzyme reaction protocol consists of four major steps: 1) 5 μL of 5× concentration of compound in 3.5% DMSO is added to each plate. 2) 10 μL of 2.5× of kinase enzyme in 1.25× kinase buffer is added and incubated for fifteen minutes at room temperature. 3) 10 μL of peptide and ATP in 1.25× kinase buffer is added to initiate the reaction. The reaction is incubated at room temperature. 4) The reaction is stopped by the addition of EDTA to a final concentration of 20 nM.

**Table 2:** Kinases in the selectivity panel

**Kinase**	**UniProt accession**
ABL	P00519
AKT	P31749
AURA	O14965
CDK2	P24941
CHK1	O14757
CKIIa	P19138
EGFR	P00533
ERK2	P28482
FGFR1	P11362
GSK3b	P49841
HGFR	P08581
IKKb	O14920
IKKi	Q14164
IRK	P06213
KDR/VEGF	P35968
LCK	P06239
MK2	P49137
MST4/MASK	Q9P289
p38	Q16539
PAK4	O96013
PDK1	O15530
PKAca	P17612
PKCb	P05771
ROCKI	Q13464
SRC	P12931
TAO3	Q9H2K8
TRKA	P04629

The kinase buffer is comprised of Hepes, a divalent cation (Mg2+ or Mn2+), and brij detergent. The concentration of each substrate was optimized for each kinase individually. Each assay is run at the K_m _concentration of ATP for the relevant kinase with an incubation time that is within the linear reaction time.

For the radiometric assays, tracer amounts of gamma ^33^P labeled ATP are included in the reaction. Once the reactions were stopped, they were transferred to Perkin Elmer Flashplates™. The plates were washed with 50 mM Hepes, and soaked for one hour in 500 μM unlabeled ATP. The plates were then re-washed with 50 mM Hepes and read in a TopCount detector.

For the Caliper mobility shift assay, after the reactions were stopped, the plates were read on a Caliper LC300 using a 12-sipper chip where separation conditions were optimized for each kinase. To measure the amount of substrate converted to product, product to sum ratios were reported.

### SB-203580

**Figure 3:**
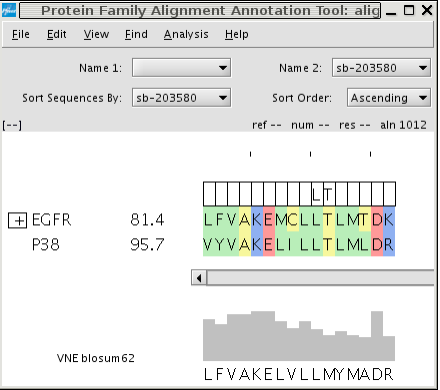
**S-Filter results for SB-203580**. S-Filter is available through the PFAAT application. Percent inhibition values are displayed to the right of the kinase name. S-Filter analysis was restricted to alignment columns that correspond to residues that make contact with SB-203580. All residues that do not make contact with SB-203580 are hidden so that all contact resides appear as a contiguous sequence. S-Filter has applied filters to the tenth and eleventh columns as indicated by the filter boxes above the respective columns. Leucine and threonine are predicted as selectivity determinants, and all uninhibited kinases that do not have these residues are hidden by the respective filters.

SB-203580 selectively inhibits EGFR and p38 in the kinase panel (Figure 2). These two kinases are on distinct branches of the kinome tree [1] and share relatively low sequence identity in the binding site. S-Filter predicts that Leu 104 and Thr 106 are specificity determinants for SB-203580 (Figure 3). SB-203580 is a well-characterized compound, and the structural basis for its selectivity at the gatekeeper position is well known [11,31]. The relatively small threonine provides access to the so-called selectivity pocket. For example, ERK2 is not inhibited by SB-203580 as it has a bulkier glutamine at this position. However, mutation of glutamine 105 to a threonine makes ERK2 susceptible to inhibition by SB-203580 [31]. These experimental observations support the prediction of Thr 106 as a specificity determinant and we designate the prediction as a true positive in Table 1.

**Figure 4:**
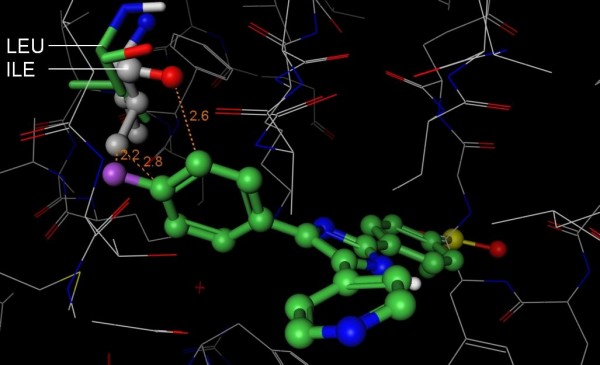
**Structural basis for SB-203580 selectivity**. The 3D structure of Abl (PDB 1M52, wire rendering with grey carbons) was superposed onto the structure of p38 (hidden) in complex with SB-203580 (PDB 1A9U, ball and stick rendering with green carbons). Like p38, Abl has a Thr at the gatekeeper position and suggests that there are other selectivity determinants that prevent SB-203580 from inhibiting Abl. The fluorophenyl of SB-203580 appears to clash (orange lines) with Ile 313 (ball and stick rendering with grey carbons) of Abl. In contrast, Leu 104 (stick rendering with green carbons) of p38 accommodates the compound. Importantly, the isoleucine is deep in the pocket where other isoleucine rotamers are likely to clash with other residues in Abl. Based on this putative steric clash, we propose that S-Filter correctly predicted Leu 104 as specificity determinant for Roscovitine.

However, three of the kinases that have a threonine at the gatekeeper position are not inhibited by SB-203580, indicating that there are additional specificity determinants. To the best of our knowledge, this is the first time that Leu 104 has been proposed as a selectivity-determining residue. Following this prediction, we explored the consequences of SRC, LCK, and ABL having an isoleucine in place of Leu 104. When ABL is superposed onto the structure of p38-SB-203580, the isoleucine of ABL appears to clash with the fluorophenyl of SB-203580 (Figure 4). In contrast, Leu 104 of p38 is free to rotate its side chain and accommodate the fluorophenyl. Based on this putative steric clash, we propose that S-Filter correctly predicted Leu 104 and we self-designate the prediction as a true positive in Table 1. This prediction demonstrates S-Filter's ability to flag selectivity determinants that have been overlooked by previous efforts. In this case, key information was derived from the multiple sequence alignment. In summary, the above observations suggest that the two predictions are correct.

### PHA-00781089

**Figure 5:**
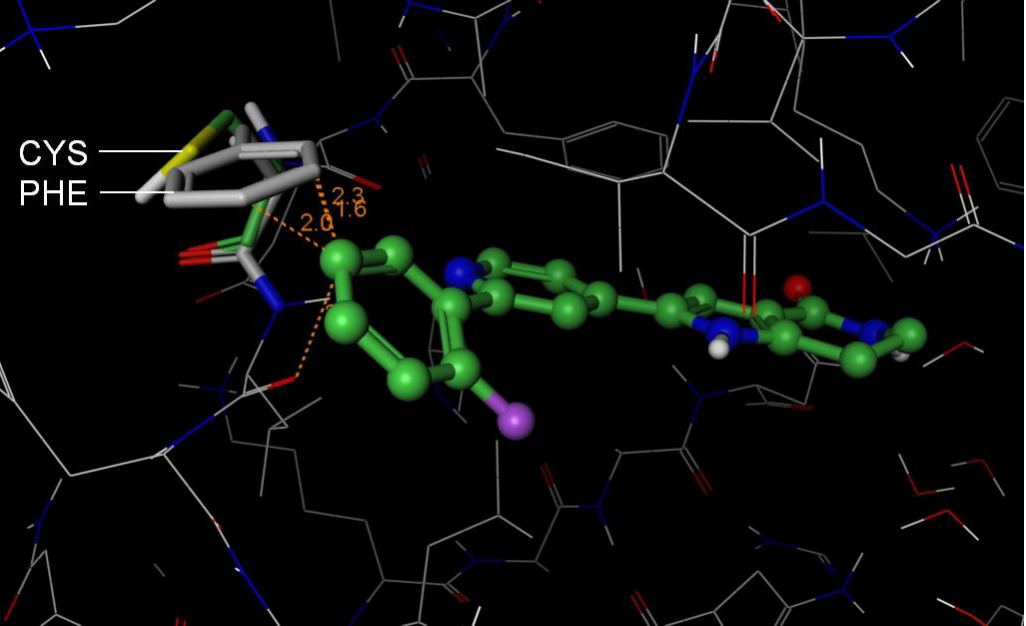
**Structural basis for PHA-00781089 selectivity**. The 3D structure of CDK2 (PDB 2A4L, wire rendering with grey carbons) was superposed onto the structure of MK2 (hidden) in complex with PHA-00781089 (PDB 2P3G, ball and stick rendering with green carbons). Anderson *et al *[22] demonstrated that the fluorophenyl of PHA-00781089 provided selectivity against CDK2 as a compound without this substituent was a potent inhibitor of CDK2. Consistent with this observation, the fluorophenyl of PHA-00781089 makes a number of clashes (orange lines) with Phe 82 (stick rendering with grey carbons) of CDK2, whereas Cys 140 (stick with green carbons) of MK2 accommodates the inhibitor. We therefore conclude that S-Filter correctly predicted Cys 140 as a specificity determinant.

PHA-00781089 selectively inhibits MK2 in the kinase panel (Figure 2). S-Filter predicts Cys 140 and Gly 143 to be specificity determinants for PHA-00781089. The structure of PHA-00781089 in complex with MK2 was recently reported [22]. Anderson *et al *demonstrated that a similar compound that lacks the fluorophenyl substituent potently inhibited CDK2, thus indicating the importance of this position for selectivity against CDK2. The fluorophenyl interacts with Cys 140 in MK2, whereas the majority of protein kinases (e.g. CDK2) have a bulkier side chain at this position. Anderson *et al *concluded that a bulkier side chain would lead to a steric clash with the fluorophenyl of PHA-00781089 (Figure 5). Therefore, the prediction of Cys 140 as specificity determinant is designated as a true positive in Table 1.

Gly 143 is at the opening of the binding site and we have no further evidence to suggest that it is a specificity determinant for PHA-00781089. This prediction is considered a false positive. Interestingly, S-Filter selected Gly 143 in its first iteration, and this selection became redundant when Cys 140 was subsequently selected. In summary, the experiments described above suggest that one of the two predictions are correct.

### Roscovitine

Roscovitine selectively inhibits CDK2 in our kinase panel (Figure 2). The purine ring of Roscovitine is unusual in that it adopts a different orientation to the purine ring of ATP. This is due to the benzyl substitution on 6-NH. Superposing the purine ring of Roscovitine onto ATP suggests that the benzyl ring of Roscovitine would clash with Phe 80 of CDK2. It is likely that all of the uninhibited kinases in the panel are able to accommodate the outward facing benzyl ring. S-Filter predicts that Val 64, Leu 83, and Ala 144 are specificity-determining residues. Unfortunately, specificity determining residues have not been experimentally determined for Roscovitine.

**Figure 6:**
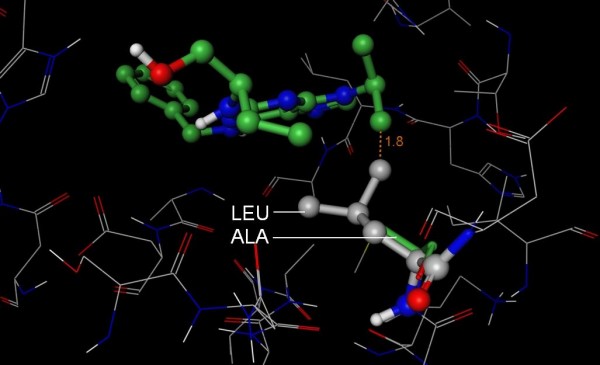
**Structural basis for Roscovitine selectivity**. The 3D structure of p38 (PDB 2GTM, wire rendering with grey carbons) was superposed onto the structure of CDK2 (hidden) in complex with Roscovitine (PDB 2A4L, ball and stick rendering with green carbons). Roscovitine appears to clash (orange lines) with Leu 167 (ball and stick rendering with grey carbons) of p38. Importantly, Leu 167 is buried deep in the pocket where there is little room to maneuver. In contrast, Ala 144 (stick rendering with green carbons) of CDK2 accommodates the compound. Based on this putative steric clash, we propose that S-Filter correctly predicted Ala 144 as specificity determinant for Roscovitine.

To the best of our knowledge, this is the first time that Ala 144 has been proposed as a selectivity-determining residue. When p38 is superposed onto CDK2, Roscovitine appears to clash with Leu 167 of p38 (Figure 6). This putative steric clash suggests that a bulkier residue in place of Ala 144 will not accommodate Roscovitine. Based on this putative steric clash, we propose that S-Filter correctly predicted Ala 144 and we self-designate the prediction as a true positive in Table 1. The remaining predictions are regarded as false positives. In summary, the above observations suggest that one of the three predictions are correct.

### PP1

**Figure 7:**
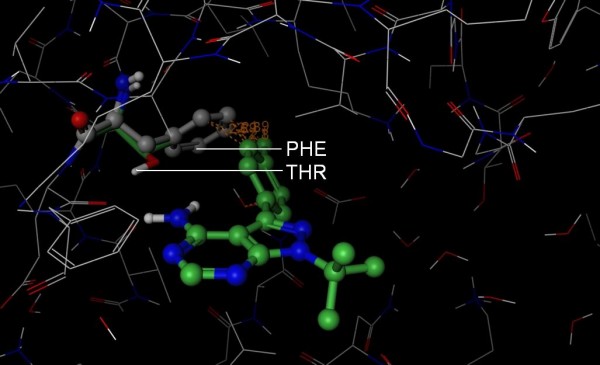
**Structural basis for PP1 selectivity**. The 3D structure of CDK2 (PDB 2A4L, wire rendering with grey carbons) was superposed onto the structure of HCK (hidden) in complex with PP1 (PDB 1QCF, ball and stick rendering with green carbons). PP1 makes a number of clashes (orange lines) with Phe 80 (ball and stick rendering with grey carbons) of CDK2 whereas Thr 338 (stick rendering with green carbons) of HCK accommodates the compound. By mutating the threonine to a bulkier residue, Liu *et el *demonstrated that this position was an important specificity determinant for PP1[32]. We therefore conclude that S-Filter correctly predicted Thr 338 as a specificity determinant.

PP1 is selectively inhibits SRC, LCK, EGFR, and ABL in our kinase panel (Figure 2). The three-dimensional structure of PP1 was solved in complex with HCK. We refer to HCK numbering in the text below. S-Filter predicts Thr 338 and Gly 344 as specificity determinants. Schindler *et al *describe Thr 338 as a specificity-determining residue for PP1 [20]. Liu *et el *demonstrated that a single residue difference at position 338 could account for the differences in potency observed between SRC and v-SRC [32]. By mutating Ile 338 to different residue types, including threonine, they demonstrated that the presence of a small residue was necessary for potent inhibition by PP1. Liu *et al *concluded that kinases with a bulkier residue at position 338 will clash with PP1 (Figure 7). These experimental observations support the prediction of Thr 338 as a specificity determinant and we designate the prediction as a true positive in Table 1.

We could not find any evidence to suggest that Gly 344 is a specificity determinant for PP1, and this prediction is designated as a false positive. However, Thr 338 cannot be the only specificity determinant, as p38 is not inhibited by PP1, despite having a threonine at the gatekeeper position. As PP1 is small and does not probe deeply into the selectivity pocket, it is possible that it does not form high-affinity interactions with the corresponding p38 residues. In summary, the experiments described above suggest that one of the two predictions are correct.

### OSI-774/CP-358774

OSI-774 selectively inhibits EGFR and ABL in our kinase panel (Figure 2). S-Filter predicts Thr 766 and Cys 773 to be selectivity determinants for OSI-774. Unfortunately, Stamos *et al *do not explicitly describe specificity determinants for OSI-774 [21].

**Figure 8:**
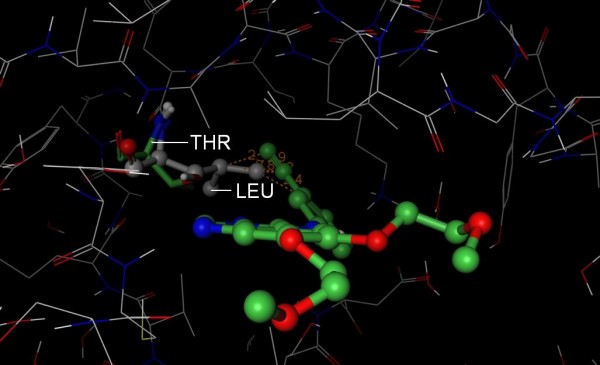
**Structural basis for OSI-774 selectivity**. The 3D structure of CHK1 (PDB 2BRH, wire rendering with grey carbons) was superposed onto the structure of EGFR (hidden) in complex with OSI-774 (PDB 1M17, ball and stick rendering with green carbons). OSI-774 appears to clash (orange lines) with Leu 84 (ball and stick rendering with grey carbons) of CHK1, whereas Thr 766 (stick rendering with green carbons) of EGFR accommodates the compound. Based on these putative steric clashes, we propose that S-Filter correctly predicted Thr 766 as a specificity determinant.

Although OSI-774 does not access the so called specificity pocket that lies beyond Thr 766, the presence of a bulkier residue may prevent inhibition. For example, when CHK1 is superposed onto EGFR, OSI-774 appears to clash with Leu 84 of CHK1 (Figure 8). This putative steric clash suggests that a bulkier residue in place of Thr 766 will not accommodate OSI-774. Based on this putative steric clash, we propose that S-Filter correctly predicted Thr 766 and we self-designate the prediction as a true positive in Table 1. As Cys 773 is at the opening of the binding site, it is not clear how it could be specificity-determining, and it is designated as a false positive. This false positive highlights the shortcomings of S-Filter and the need to carefully assess all predictions before proceeding. In summary, the above observations suggest that one of the two predictions is correct.

### GW-572016

**Figure 9:**
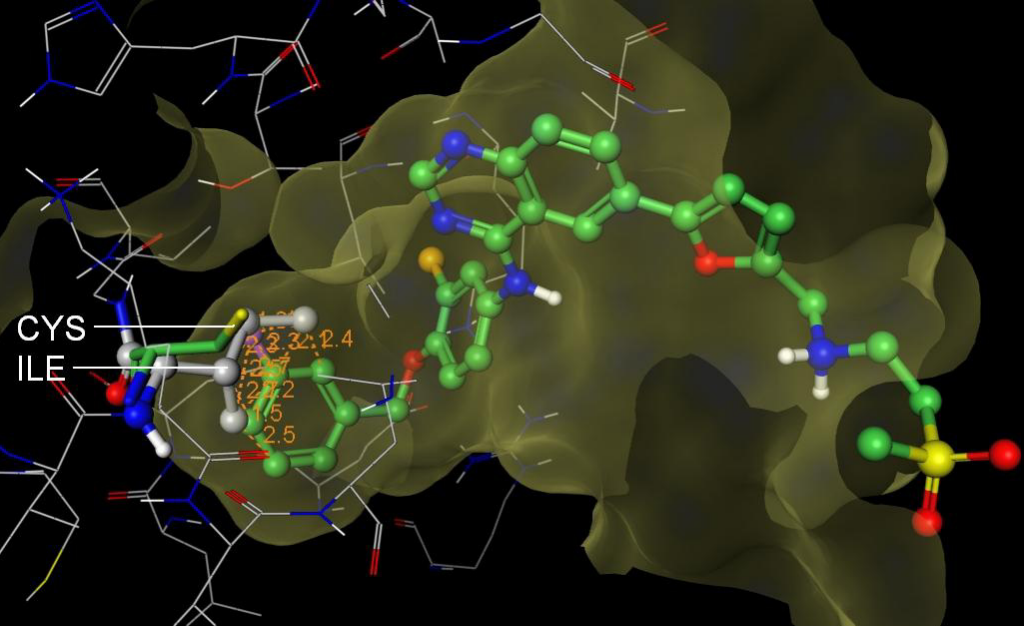
**Structural basis for GW-572016**. The 3D structure of p38 (PDB 1A9U, wire rendering with grey carbons) was superposed onto the structure of EGFR (hidden) in complex with GW-572016 (PDB 1XKK, ball and stick rendering with green carbons). GW-572016 appears to clash (orange lines) with Ile 84 (ball and stick rendering with grey carbons) of p38. In contrast, Cys 775 (stick rendering with green carbons) of EGFR accommodates the compound. Importantly, Ile 84 is buried deep in the protein where there is little room to maneuver and clearly protrudes the pocket (mustard) of EGFR. Based on this putative steric clash, we propose that S-Filter correctly predicted Cys 775 as specificity determinant for GW-572016.

GW-572016 selectively inhibits EGFR in our kinase panel (Figure 2). S-Filter predicts Cys 775, as a specificity determinant for GW-572016. Wood *et al *do not explicitly describe specificity determinants for GW-572016[23]. To the best of our knowledge, this is the first time that Cys 775 has been proposed as a selectivity-determining residue. When p38 is superposed onto EGFR, GW-572016 appears to clash with Ile 84 of p38 (Figure 9). This putative steric clash suggests that many of the kinases in our panel with a bulkier residue in place of Cys 775 will not accommodate GW-572016. In contrast, cysteine has a non-branched side chain that presumably allows it to rotate and accommodate GW-572016. Based on this putative steric clash, we propose that S-Filter correctly predicted Cys 775 and we self-designate the prediction as a true positive in Table 1

S-Filter failed to predict Thr 790, which provides access to the selectivity pocket. S-Filter also failed to predict Met 766 which may provide additional specificity through its flexible side chain. Additionally, the C helix of EGFR has moved away from the binding site to accommodate GW-572016, suggesting that selectivity extends to other parts of the protein that do not directly contact GW-572016. As S-Filter does not consider conformational changes, it is recommended that S-Filter analysis is combined with structural comparisons whenever possible. In summary, the above observations suggest that S-Filter correctly predicted one specificity determinant, but also failed to predict two other specificity determinants.

### Fasudil

**Figure 10:**
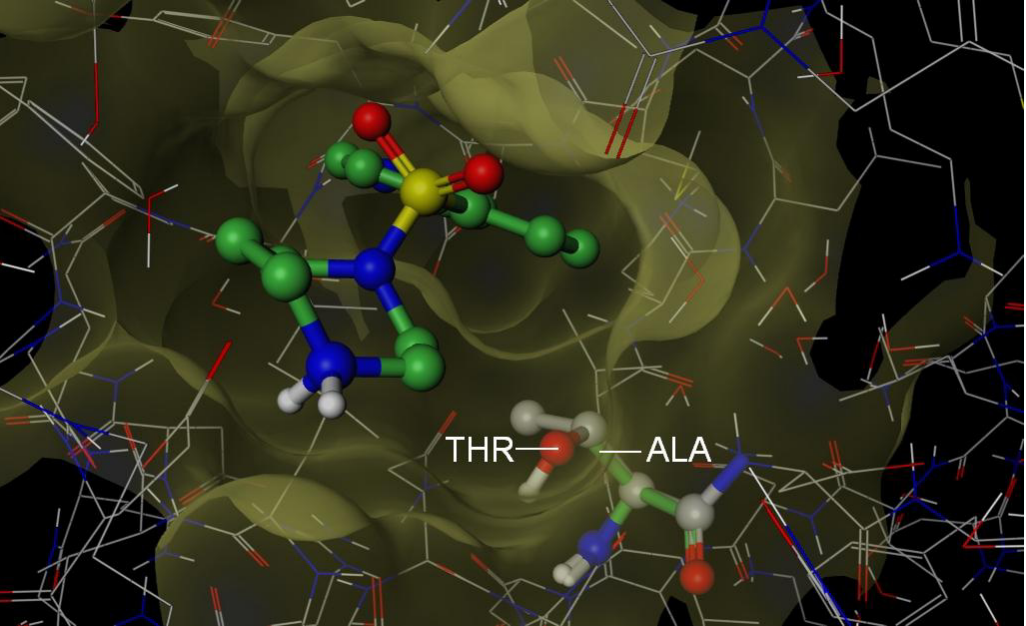
**Structural basis for Fasudil selectivity**. The 3D structure of PKA (PDB 1Q8T, wire rendering with grey carbons) superposed onto the structure of Rock1 (hidden) in complex with Fasudil (PDB 2ESM, ball and stick rendering with green carbons). Ala 215 (Stick rendering with green carbons) of Rock1 is smaller than Thr 183 (ball and stick rendering with grey carbons) of PKA which protrudes the pocket of ROCK (mustard surface). By mutating Thr 183 in PKA to the corresponding position in Rock (Ala 215), Bonn *et al *demonstrated that Fasudil inhibited the mutant PKA at levels similar to ROCK [26]. The smaller residue was proposed by Bonn *et al *to allow Fasudil deeper access to the active site. Based on the above observations, we conclude that S-Filter correctly predicted Ala 215 as a specificity determinant.

Fasudil selectively inhibits ROCK1 in our kinase panel (Figure 2). Structural and mutational studies implicate Ala 215 along with Ile 82 as selectivity determinants [24,26]. Bonn *et al *demonstrated that Fasudil is not a potent inhibitor of PKA. By mutating Thr 183 in PKA to the corresponding position in Rock (Ala 215), they demonstrated that Fasudil inhibited the mutant PKA at levels similar to ROCK. Similarly, by mutating Leu 49 in PKA to the corresponding residue in ROCK (Ile 82), they observed inhibition levels similar to wild type ROCK. Figure 10 suggests a slightly bulkier threonine in place of Ala 215 could prevent Fasudil from binding deep in the active site. Based on these experimental observations, we designate Ala 215 as a true positive in Table 1. S-Filter failed to predict Ile 82 and we designate it as a false negative in Table 1. S-Filter also predicted Val 137 and Met 153 as specificity determinants for Fasudil. We designate both of these predictions as false positives. In summary, the above observations suggest that one of the three predictions are correct.

## Discussion and Conclusion

S-filter is a hypothesis generation tool that flags potential selectivity determinants for further structural analysis or experimentation. The method relies on selectivity data, a multiple sequence alignment and parameters derived from the three dimensional structure of a kinase in complex with the compound of interest. Unlike similarity metrics that summarize multiple residue positions with a single metric (e.g. percent identity), S-Filter evaluates each residue position independently. This is important as specificity can often be attributed to a single-residue difference. By incorporating solvent accessibility data from the crystal structure, S-Filter tends to favor the prediction of specificity determinants that are deep in the pocket of the active site. This reduces the likelihood of S-Filter converging towards residue positions that are solvent exposed and less likely to be specificity determinants.

Analysis is restricted to residues that make contact with the compound, and S-Filter does not consider other residues that may exert their effects indirectly or through conformational changes, e.g. the DFG loop. Alternative binding modes [33] are not explicitly modeled, and at best S-Filter could only predict multiple specificity determinants for such cases. The presence of key interacting waters will not be explicitly taken into account. However, it is conceivable that S-Filter could detect a residue that indirectly interacts with the compound via a specific water-mediated hydrogen bond. S-Filter uses a greedy algorithm to filter for specificity determinants and has the potential to commit to a path that does not lead to the optimal prediction. The predictions made for Roscovitine and OSI-774 reveal some of the pitfalls associated with S-Filter. Fortunately, these predictions were easily dismissed after inspecting the structure.

Despite these potential shortcomings, S-Filter made a number of useful predictions that either agreed with prior experimental data or were supported by structural superposition studies. Prior experimental data was available for four of the seven compounds, and in each case S-Filter correctly predicted at least one specificity determinant (Table 1). S-Filter also made a number of novel predictions for SB-203580, Roscovitine, OSI-774 and GW-572016. These predictions point to potential selectivity determinants that appear plausible and await experimental validation. Furthermore, these predictions demonstrate the ability of S-Filter to find subtle trends that are not readily detected. Overall, eight of the fifteen predictions are regarded as true positives. However, it is important that these metrics are not interpreted as an indicator of prediction accuracy, as our validations are limited to a small number of compounds. Nevertheless, the predictions are encouraging, as the challenges associated with rationalizing inhibitor selectivity are both difficult and time-consuming. S-Filter assists this process by prioritizing residues for further structural analysis and follow-up. In instances where the selectivity can be rationalized, one might consider mutagenesis studies or compound modifications to further optimize or abrogate selectivity.

## Authors' contributions

DRC conceived and implemented the algorithm, designed the study, evaluated the predictions, and contributed to the writing of the manuscript. EAL proposed a filtering approach, evaluated the predictions, and contributed to the writing of the manuscript. DJM performed the kinase assays and contributed to the writing of the manuscript. All authors read and approved the final manuscript.

## Supplementary Material

Additional file 1 **This file provides kinase percent inhibition values for all compounds.** Click here for file

## References

[B1] Manning G, Whyte DB, Martinez R, Hunter T, Sudarsanam S (2002). The protein kinase complement of the human genome. Science.

[B2] Cohen P (2002). Protein kinases – the major drug targets of the twenty-first century?. Nat Rev Drug Discov.

[B3] Manley PW, Cowan-Jacob SW, Buchdunger E, Fabbro D, Fendrich G, Furet P, Meyer T, Zimmermann J (2002). Imatinib: a selective tyrosine kinase inhibitor. Eur J Cancer.

[B4] Widakowich C, de Castro G, de Azambuja E, Dinh P, Awada A (2007). Review: side effects of approved molecular targeted therapies in solid cancers. Oncologist.

[B5] Castoldi RE, Pennella G, Saturno GS, Grossi P, Brughera M, Venturi M (2007). Assessing and managing toxicities induced by kinase inhibitors. Curr Opin Drug Discov Devel.

[B6] Vieth M, Higgs RE, Robertson DH, Shapiro M, Gragg EA, Hemmerle H (2004). Kinomics-structural biology and chemogenomics of kinase inhibitors and targets. Biochim Biophys Acta.

[B7] Vieth M, Sutherland JJ, Robertson DH, Campbell RM (2005). Kinomics: characterizing the therapeutically validated kinase space. Drug Discov Today.

[B8] Sheinerman FB, Giraud E, Laoui A (2005). High affinity targets of protein kinase inhibitors have similar residues at the positions energetically important for binding. J Mol Biol.

[B9] Vulpetti A, Bosotti R (2004). Sequence and structural analysis of kinase ATP pocket residues. Farmaco.

[B10] Chuaqui C, Deng Z, Singh J (2005). Interaction profiles of protein kinase-inhibitor complexes and their application to virtual screening. J Med Chem.

[B11] Wang Z, Canagarajah BJ, Boehm JC, Kassisa S, Cobb MH, Young PR, Abdel-Meguid S, Adams JL, Goldsmith EJ (1998). Structural basis of inhibitor selectivity in MAP kinases. Structure.

[B12] Karaman MW, Herrgard S, Treiber DK, Gallant P, Atteridge CE, Campbell BT, Chan KW, Ciceri P, Davis MI, Edeen PT (2008). A quantitative analysis of kinase inhibitor selectivity. Nat Biotechnol.

[B13] Taylor RD, Jewsbury PJ, Essex JW (2002). A review of protein-small molecule docking methods. J Comput Aided Mol Des.

[B14] Caffrey DR, Dana PH, Mathur V, Ocano M, Hong EJ, Wang YE, Somaroo S, Caffrey BE, Potluri S, Huang ES (2007). PFAAT version 2.0: a tool for editing, annotating, and analyzing multiple sequence alignments. BMC Bioinformatics.

[B15] Lee B, Richards FM (1971). The interpretation of protein structures: estimation of static accessibility. J Mol Biol.

[B16] Bain J, Plater L, Elliott M, Shpiro N, Hastie CJ, McLauchlan H, Klevernic I, Arthur JS, Alessi DR, Cohen P (2007). The selectivity of protein kinase inhibitors: a further update. Biochem J.

[B17] Davies SP, Reddy H, Caivano M, Cohen P (2000). Specificity and mechanism of action of some commonly used protein kinase inhibitors. Biochem J.

[B18] Fabian MA, Biggs WH, Treiber DK, Atteridge CE, Azimioara MD, Benedetti MG, Carter TA, Ciceri P, Edeen PT, Floyd M (2005). A small molecule-kinase interaction map for clinical kinase inhibitors. Nat Biotechnol.

[B19] De Azevedo WF, Leclerc S, Meijer L, Havlicek L, Strnad M, Kim SH (1997). Inhibition of cyclin-dependent kinases by purine analogues: crystal structure of human cdk2 complexed with roscovitine. Eur J Biochem.

[B20] Schindler T, Sicheri F, Pico A, Gazit A, Levitzki A, Kuriyan J (1999). Crystal structure of Hck in complex with a Src family-selective tyrosine kinase inhibitor. Mol Cell.

[B21] Stamos J, Sliwkowski MX, Eigenbrot C (2002). Structure of the epidermal growth factor receptor kinase domain alone and in complex with a 4-anilinoquinazoline inhibitor. J Biol Chem.

[B22] Anderson DR, Meyers MJ, Vernier WF, Mahoney MW, Kurumbail RG, Caspers N, Poda GI, Schindler JF, Reitz DB, Mourey RJ (2007). Pyrrolopyridine inhibitors of mitogen-activated protein kinase-activated protein kinase 2 (MK-2). J Med Chem.

[B23] Wood ER, Truesdale AT, McDonald OB, Yuan D, Hassell A, Dickerson SH, Ellis B, Pennisi C, Horne E, Lackey K (2004). A unique structure for epidermal growth factor receptor bound to GW572016 (Lapatinib): relationships among protein conformation, inhibitor off-rate, and receptor activity in tumor cells. Cancer Res.

[B24] Jacobs M, Hayakawa K, Swenson L, Bellon S, Fleming M, Taslimi P, Doran J (2006). The structure of dimeric ROCK I reveals the mechanism for ligand selectivity. J Biol Chem.

[B25] Daylight Chemical Information System Inc. 120 Vantis – Aliso Viejo, CA 92656.

[B26] Bonn S, Herrero S, Breitenlechner CB, Erlbruch A, Lehmann W, Engh RA, Gassel M, Bossemeyer D (2006). Structural analysis of protein kinase A mutants with Rho-kinase inhibitor specificity. J Biol Chem.

[B27] Johnson M, Li C, Rasnow B, Grandsard P, Xing H, Fields A (2002). Converting a Protease Assay to a Caliper Format LabChip System. Journal of the Association for Laboratory Automation.

[B28] Dunne J, Reardon H, Trinh V, Li E, Farinas J (2004). Comparison of On-Chip and Off-Chip Microfluidic Kinase Assay Formats. Assay Drug Dev Technol.

[B29] Schnurr B, Schächtele C Use of FlashPlate for Automated Kinase Assays. Perkin Elmer Application Note, FlashPlate^® ^File #6. http://www.perkinelmer.com/lifesciences.

[B30] Hastie CJ, McLauchlan HJ, Cohen P (2006). Assay of protein kinases using radiolabeled ATP: a protocol. Nat Protoc.

[B31] Fox T, Coll JT, Xie X, Ford PJ, Germann UA, Porter MD, Pazhanisamy S, Fleming MA, Galullo V, Su MS (1998). A single amino acid substitution makes ERK2 susceptible to pyridinyl imidazole inhibitors of p38 MAP kinase. Protein Sci.

[B32] Liu Y, Bishop A, Witucki L, Kraybill B, Shimizu E, Tsien J, Ubersax J, Blethrow J, Morgan DO, Shokat KM (1999). Structural basis for selective inhibition of Src family kinases by PP1. Chem Biol.

[B33] De Moliner E, Brown NR, Johnson LN (2003). Alternative binding modes of an inhibitor to two different kinases. Eur J Biochem.

